# The Effects of Arbuscular Mycorrhizal Fungal Inoculation and Mulch of Contrasting Chemical Composition on the Yield of Cassava under Humid Tropical Conditions

**DOI:** 10.1100/tsw.2010.59

**Published:** 2010-04-01

**Authors:** Iniobong E. Okon, M. G. Solomon, O. Osonubi

**Affiliations:** ^1^ Department of Botany, University of Calabar, Calabar, Nigeria; ^2^ Department of Soil Science, University of Calabar, Calabar, Nigeria; ^3^ Department of Botany and Microbiology, University of Ibadan, Ibadan, Nigeria

**Keywords:** cassava, arbuscular mycorrhiza, green mulch, yield

## Abstract

The influence of arbuscular mycorrhizal fungus (AMF), *Glomus deserticola*, and leaf mulch from *Gliricidia sepium* and Senna siamea on the yield of cassava (*Manihot esculenta*) in a degraded alfisol of southwestern Nigeria was investigated. Inoculation in conjunction with mulching increased cassava tuber yield by 40–278% over the control. The highest yield was obtained with *G. sepium* and *S. siamea* mulch applied together in equal proportions. The results are explained in the light of the growth-enhancing effects of AMF, encouraged by the ameliorating effects of mulch on the soil structure and nutrient contents.

### INTRODUCTION

Arbuscular mycorrhizal fungi (AMF) are widely occurring soil microorganisms that form symbiotic association with the roots of higher plants, resulting in a variety of effects on the growth of the host plant. They have been known to enhance crop growth and yield[1] through increased water and nutrient uptake, as well as alteration of some physiological processes in the plants that result in increased yield[2].

Cassava is an important staple food in tropical Africa. It is mostly grown by resource-poor farmers[3]. Its high-nutrient requirements and the resulting nutrient-extraction capacity, especially potassium[4], limit yield and make its production insufficient in most areas with limited farmland. To offset subsequent years' poor tuber yield, nutrients must be added to replenish those that have been lost through tuber and stem harvesting. Inorganic fertilizers have been used to increase tuber yield in cassava[5], but the resource-poor farmers cannot afford them due to high cost. Viable alternative and cheap nutrient sources can be obtained from green mulch.

Cassava is highly mycorrhizal dependent for nutrient extraction[4,6]. Inoculation with appropriate AMF can enhance the efficiency of cassava roots to extract nutrients released by the mulch. Mulch on its own has favorable effects on the physical, chemical, and biological properties of the soil, as well as enhanced root development, all of which lead to improved crop performance and yield[7,8].

This study was conducted to determine the influence of mulch of varying chemical composition and inoculation with *Glomus deserticola* on the yield of cassava genotype TMS 4(2)1425.

### MATERIALS AND METHODS

#### Site Description

This field experiment was conducted at the research farm of the University of Ibadan, Ibadan, Nigeria (7° 43' N, 3° 9' E). The soil of the experimental site is classified as a Rhodic Kandiustalf (Balogun series) with the following surface (0–15 cm) soil properties as obtained by analyses using Juo's[9] method of soil analysis: organic C, 0.98%; total N, 0.036%; pH 6.50; CEC 5.63 cmol $kg^{-1}$; Ca, 3.12 mg $kg^{-1}$; Mg, Na, K, 1.19, 0.11, and 0.19 cmol $kg^{-1}$, respectively; while the available P was 1.17 mg $kg^{-1}$ as obtained using Bray and Kurtz's[10] method and read on an electrophotometer at 660-mu wavelength[9]. The total rainfall for the period of the experiment was 1298.4 mm, while the total evaporation was 1499.02 mm.

#### Experimental Design and Layout

The experiment was conducted with treatments laid out in a split-plot design and each replicated thrice. The main plots consisted of AMF *G. deserticola*, inoculation arranged in blocks. Treatments were split on mulch type as subplots. Each subplot plot was 12 × 12 m in area. The experimental treatments consisted of AMF inoculated (*Gd*+) and uninoculated (*Gd*-), *Gliricidia sepium* (C:N ratio of 8), *Senna siamea* (C:N ratio of 13), and *G. sepium* + *S. siamea* (1:1) mixture mulch. *G. sepium* is a nodulating legume of the Papilionaceae family, while *S. siamea* is a non-nodulating legume of the Caesalpiniaceae family.

Cassava (*Manihot esculenta* Crantz) cv TMS 4(2)1425 was planted on ridges at a spacing of 1 m apart and 1 m within the rows on ridges to give a population of 10,000 cassava plants per hectare. Mycorrhizal plots were inoculated with *Glomus deserticola* Trappe, Bloss, and Menge (INVAM, CA 113). Inoculation was done by applying 20-g crude inoculum of *G. deserticola* (containing approximately 440–610 spores) in the planting holes of the cassava stem cuttings (0.2 m long) before covering with soil. At the beginning of the experiment, 0.5 t $ha^{-1}$ dry matter equivalent of green mulch from *Gliricidia sepium*, *S. siamea*, or a mixture of both in a 1:1 ratio were applied. Subsequent mulching at 2-month intervals was applied at the same rate of 0.5 t $ha^{-1}$ dry matter to their respectively designated subplots. A basal fertilizer dressing of 25 kg $ha^{-1}$ (NPK 27:10:10) was applied at 3 months after planting. Weeding was done manually, when necessary, throughout the growing season.

Harvesting was done at 12 months after planting. The fresh weight of root tubers was measured, while the dry weight of stems and leaves was taken after oven drying to constant weight at 70°C. Subsamples of tubers were oven dried and ground for N, K, and P content analyses following Juo's[9] method for plant analysis. The uptake of the nutrients was calculated as the product of percentage nutrient concentration and yield[8].

Percentage AMF colonization of roots was determined after clearing and staining them with trypan blue using the procedure of Koske and Gemma[11] and viewing under a stereomicroscope at ×45 magnification on a gridline plate[12].

Samples of mulch used from each plant species were pooled together and analyzed for N, P, and K contents after Juo's[9] method.

All data were subjected to a combined analysis of variance (ANOVA) procedure using the Windows version of Statistical Analysis System[13], with vesicular AMF treatment as the main plots and mulch type as subplots. Mean values were compared using Duncan's Multiple Range Test (DMRT) at 0.05 level of probability when the F-ratio was significant.

## RESULTS

### AMF Colonization

Cassava roots were all colonized by AMF (Table 1). Roots from inoculated plants, however, showed a significantly higher percentage of AMF colonization than those of uninoculated plants. Colonization was also higher in mulched plants than the unmulched, except for *S. siamea,* which was lower than the inoculated control. Colonization was found to be highest in *G. sepium* and *S. siamea* mixed-mulch inoculated plants and lowest in unmulched control.

**TABLE 1**
**Effect of *Glomus deserticola* and Mulching on AMF Colonization and Yield of Cassava**

| Mulch | Inoculation | AMF Colonization (%) | Leaf, Dry Weight | Stem, Dry Weight | Fresh Tuber Weight |
|---|---|---|---|---|---|
| | | | | (t ha$^{-1}$) | |
| *S. siamea* | *Gd+* | 53.3 ± 3.3$^{d}$ | 1.4 ± 0.0$^{d}$ | 1.6 ± 0.1$^{d}$ | 12.3 ± 0.4$^{c}$ |
| | *Gd–* | 25.0 ± 1.3$^{f}$ | 0.4 ± 0.0$^{f}$ | 0.4 ± 0.0$^{f}$ | 5.0 ± 0.4$^{e}$ |
| *G. sepium* | *Gd+* | 81.5 ± 1.9$^{b}$ | 5.0 ± 0.0$^{b}$ | 7.4 ± 0.2$^{b}$ | 20.0 ± 1.4$^{b}$ |
| | *Gd–* | 33.7 ± 4.5$^{e}$ | 2.2 ± 0.0$^{c}$ | 2.6 ± 0.1$^{c}$ | 7.5 ± 1.1$^{e}$ |
| *Gs + Ss* | *Gd+* | 86.7 ± 2.1$^{a}$ | 5.8 ± 0.4$^{a}$ | 8.5 ± 0.3$^{a}$ | 33.3 ± 1.3$^{a}$ |
| | *Gd–* | 59.9 ± 3.3$^{c}$ | 2.5 ± 0.1$^{c}$ | 2.5 ± 0.5$^{c}$ | 8.3 ± 0.3$^{d}$ |
| Control | *Gd+* | 60.4 ± 2.0$^{c}$ | 0.7 ± 0.1$^{e}$ | 1.5 ± 0.3$^{d}$ | 8.8 ± 0.9$^{d}$ |
| | *Gd–* | 18.7 ± 2.8$^{g}$ | 0.4 ± 0.0$^{ef}$ | 0.6 ± 0.0$^{e}$ | 5.7 ± 0.2$^{f}$ |

Values are means of three replicates ± S.E. Means in the same column followed by different letters are significantly different at $p < 0.05$ according to Duncan's multiple range test. *Gs + Ss*, *Gliricidia sepium* and *S. siamea* mixed mulch; *Gd+*: *Glomus deserticola* inoculated; *Gd–*: *G. deserticola* uninoculated.

### Biomass Yield

All the yield components of cassava measured were significantly increased by inoculation and mulching (Table 1). The only exceptions to this were uninoculated *S. siamea* mulched plants whose biomass yields were significantly lower than unmulched inoculated controls. Applying a mixture of *Gliricidia sepium* and *S. siamea* mulch in all cases gave the highest leaf and stem dry matter as well as root tuber yields for both inoculated and uninoculated cassava plants.

### Cassava Root Tuber Nutrient Yield

Tuber nutrient yield for N, P, and K were significantly higher in all inoculated plants than uninoculated plants (Table 2). Mulching also increased tuber nutrient yield, except for senna-mulched plants.

### Nutrient Contributions from Mulching Materials

Analyses for N, P, and K content of the three mulch treatments used showed that nitrogen and potassium contribution by the mulch were highest from *Gliricidia sepium* followed by the *G. sepium* + *S. siamea*

**TABLE 2**
**Effect of *Glomus deserticola* and Mulch on the Tuber Nutrient Uptake of Cassava**

| Mulch | Inoculation | N | P | K |
|---|---|---|---|---|
| | | (kg ha$^{-1}$) | | |
| *S. siamea* | *Gd+* | 11.3 ± 0.9$^{d}$ | 3.3 ± 0.3$^{c}$ | 22.3 ± 1.8$^{c}$ |
| | *Gd–* | 5.4 ± 0.6 | 1.2 ± 0.2$^{f}$ | 6.8 ± 0.8$^{f}$ |
| *G. sepium* | *Gd+* | 38.7 ± 1.9$^{b}$ | 5.0 ± 0.2$^{b}$ | 29.5 ± 1.5$^{b}$ |
| | *Gd–* | 8.9 ± 0.2$^{b}$ | 1.4 ± 0.0$^{f}$ | 10.5 ± 0.2$^{e}$ |
| *Gs + Ss* | *Gd+* | 58.2 ± 4.2$^{a}$ | 8.1 ± 0.6$^{a}$ | 114.5 ± 8.2$^{a}$ |
| | *Gd–* | 15.3 ± 0.8$^{c}$ | 2.7 ± 0.2$^{d}$ | 15.5 ± 0.8$^{d}$ |
| Control | *Gd+* | 14.2 ± 1.1$^{c}$ | 1.9 ± 0.1$^{e}$ | 11.2 ± 0.9$^{e}$ |
| | *Gd–* | 8.2 ± 0.2$^{e}$ | 1.3 ± 0.2$^{f}$ | 12.3 ± 1.9$^{e}$ |

Values are means of three replicates ± S.E. Means in the same column followed by different letters are significantly different at $p < 0.05$ according to Duncan's multiple range test. *Gs + Ss*, *Gliricidia sepium* and *S. siamea* mixed mulch; *Gd+*: *Glomus deserticola* inoculated; *Gd–*: *G. deserticola* uninoculated.

**TABLE 3**
**Total N, P, and K Contributed to the Soil by the Applied Mulch**

| Mulch | N | P | K |
|---|---|---|---|
| | (kg ha$^{-1}$) | | |
| *S. siamea* | 68.4 ± 6.3$^{b}$ | 4.5 ± 0.3$^{bc}$ | 23.3 ± 3.1$^{b}$ |
| *G. sepium* | 102.8 ± 15.5$^{a}$ | 4.9 ± 1.2$^{b}$ | 43.6 ± 5.7$^{a}$ |
| *Gs + Ss* | 91.8 ± 13.8$^{a}$ | 6.3 ± 1.6$^{a}$ | 37.8 ± 6.4$^{a}$ |

Values are means of three replicates ± S.E. Means in the same column followed by different letters are significantly different at $p < 0.05$ according to Duncan's multiple range test. *Gs + Ss*, *Gliricidia sepium* and *S. siamea* mixed mulch.

mixture, while *S. siamea* contributed the lowest. Phosphorus on the other hand was contributed significantly highest from *G. sepium* + *S. siamea* mixed mulch followed by *G. sepium* and *S. siamea*, respectively (Table 3).

### DISCUSSION

Cassava roots from inoculated plots had significantly higher AMF colonization than those from uninoculated plots. This shows that the introduced *Glomus deserticola* was more efficient in colonization than the indigenous mycorrhizal fungi. Besides, it is possible that the population of viable indigenous AMF propagules available to the plants was extremely low since the plot had been under continuous cropping without any mycorrhizal management. *Glomus mosseae* (Nicholson and Gerdemann),

*Acaulospora scrobiculata* (Trappe), and *A. spinosa* (Walker and Trappe) were the AMF isolated using Daniels and Skipper's[14] method and identified[15] from the experimental site. Although there are no records of their being less effective than *G. deserticola*, from our infective AMF propagule estimation results using Sieverding's[16] method, the infective propagules and spores per 100-g dry soil were significantly higher in all inoculated plots.

Higher colonization in mulched plots lends support to Saif's[17] findings that addition of crop residues to soil increases AMF colonization. It appears as if the mulch conserved moisture and nutrients, thus making the soil conditions conducive for the AMF colonization. In addition, Cavender et al.[18] have shown that nutrients present in vermicompost stimulated fungal colonization. This is further supported by the fact that a mixture of *Gliricidia sepium* + *S. siamea* mulch, which covered the soil surface better and decomposed slower, gave the highest AMF colonization of cassava roots. The effect of organic matter on AMF colonization has, however, been suggested to be an indirect one involving the organic matter effect on the soil structure, water holding capacity, and nutrient mineralization[19].

The increase of cassava yield components by AMF inoculation is to be expected, since AMF is known to increase the growth yield of plants[2,20]. These they do through increased net photosynthesis[21], and enhanced water and nutrient uptake[22,23,24]. This enhanced nutrient uptake explanation is supported by the result of nutrient analysis (Table 2). Other possible reasons for the increased yield may include modification of host hormonal relations[25] and modification of soil structure[26], all of which will result in favorable conditions for growth and yield. The implication of inoculation with higher yield is best illustrated by a trend of relationship between AMF colonization and the yield components in Table 1.

Mulching significantly increased all the yield components of cassava, especially when in conjunction with AMF inoculation. This can be attributed to the addition of nutrients to the soil as well as amelioration of soil physical properties by the mulch[27]. The similarity in yield between inoculated unmulched and uninoculated mulched in the *G. sepium* as well as *G. sepium* + *S. siamea* mulch-mixture treatments indicated the possibility of a growth-enhancing effect resulting from the *Glomus deserticola* reaching out beyond the nutrient depletion zone to extract nutrients for its host. *S. siamea* mulch alone, however, gave the lowest yield in all cases of mulch application. This suggests the unsuitability of *S. siamea* mulch alone for cassava cropping, but that advantage can be taken of its slow decomposition and gradual nutrient release to combine it with the fast decomposing *Gliricidia sepium* mulch for maximum yield of cassava. Vinod and Nair[28] reported a positive effect of slow release of nitrogenous fertilizers on the growth and yield of cassava. Being a slow-growing crop, cassava will then derive the benefit of adequate time to draw from the decomposing mulch without much nutrient loss through leaching. The highest biomass yield from inoculated and mulched plants indicates a positive synergistic effect of these two factors, which can be manipulated to ensure increased cassava tuber production. Vaidya et al.[29] demonstrated a strong positive influence of organic matter on AMF proliferation. The higher nutrient uptake of N, P, and K in inoculated and mulched plants over the control can be explained through additional nutrients to the soil and increased P uptake by the crop[30], which in turn increased root growth and development, a condition capable of increasing the uptake of other nutrients and water.

### CONCLUSION

The cassava genotype TMS 4(2)1425 used in this study responded positively to *Glomus deserticola* inoculation in conjunction with a mixture of *Gliricidia sepium* + *Senna siamea* mulch. Advantages of these findings can be used by resource-poor cassava farmers in the tropics to boost their tuber production, especially in recent times when cassava has been called a "wonder" crop in Africa due to its versatile uses for food, starch, flour, fuel, and ethanol production.

### ACKNOWLEDGMENTS

The authors are grateful to Dr. Pat Millner of USDA-ARS, Beltsville, MD for *Glomus deserticola*. The technical assistance of IITA, Ibadan, Nigeria is also gratefully acknowledged.
